# Early Bilateral Cystoid Macular Oedema Secondary to Fingolimod in Multiple Sclerosis

**DOI:** 10.1155/2012/134636

**Published:** 2012-09-13

**Authors:** Lei Liu, Fiona Cuthbertson

**Affiliations:** Department of Ophthalmology, Royal United Hospital Bath, Bath BA1 3NG, UK

## Abstract

We report a case of fingolimod-associated bilateral cystoid macular oedema in a patient with multiple sclerosis (MS). A 34-year-old female, diagnosed with MS at age of 30, developed bilateral blurred vision 5 days after initiation of fingolimod. She was misdiagnosed as optic neuritis initially and fingolimod was only discontinued 3 weeks after onset of her visual symptoms when OCT showed prominent bilateral cystoid macular oedema and subretinal fluid. Although her left corrected vision returned to 6/6, she had persistently decreased right visual acuity of 6/12 after 5 months. This paper aims to raise awareness among ophthalmologists and neurologists of the importance of early recognition of macular oedema associated with fingolimod.

## 1. Introduction

Multiple sclerosis (MS) is the most common demyelinating disease of the central nervous system affecting 2.5 million people worldwide and is the primary cause of neurological disability of young adults. In Europe, the total estimated prevalence rate of MS is 83 per 100,000 and the estimated mean annual MS incidence rate is 4.3 cases per 100,000 [1]. The major hypothesis of MS pathogenesis is autoimmunity or oligodendrogliopathy. The former is the most widely accepted hypothesis, which describes that the naïve myelin antigen-specific CD4 positive T cells primed in the peripheral lymph nodes migrate into the CNS leading to demyelination and axonal degeneration [2]. Fingolimod (FTY720, Gilenya, Novartis) is the first oral treatment approved by the US Food and Drug Administration for the treatment of relapsing-remitting multiple sclerosis (RRMS). Fingolimod is rapidly phosphorylated to form fingolimod-phosphate *in vivo*, which resembles a natural extracellular lipid mediator, sphingosine 1-phosphate (S1P), and induces internalization and degradation of S1P receptors. S1P receptor subtype 1 (S1P_1_) is expressed on lymphocytes and plays a key role in regulation lymphocyte egress from lymphoid tissues into the circulation. Fingolimod is thought to retain lymphocytes in lymphoid tissues, thus reduce the infiltration of autoaggressive lymphocytes into the CNS [3]. The TRANSFORMS study compared the efficacy of oral fingolimod (0.5 mg or 1.25 mg daily) with intramuscular interferon beta-1a (30 *μ*g weekly) on MS. At 12 months the relapse rate was significantly lower in both fingolimod groups than in the interferon beta-1a group [4]. Another study (FREEDOMS) showed a significantly reduced relapse rate of MS at 24 months with fingolimod compared to placebo [5]. Macular oedema is a known complication of fingolimod with incidence of about 0.5% with 0.5 mg oral dose [6]. 

## 2. Case Presentation

**Figure 1:**
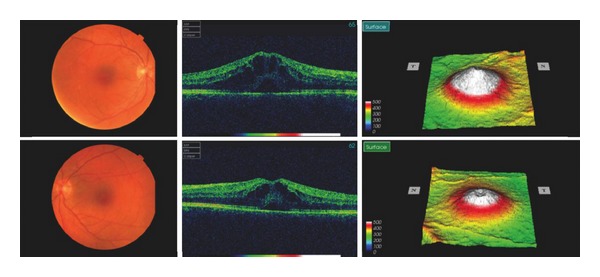
Bilateral cystoid macular oedema was prominent with subretinal fluid at 20 days after initiation of fingolimod. Right eye was worse compared with left eye.

**Figure 2:**
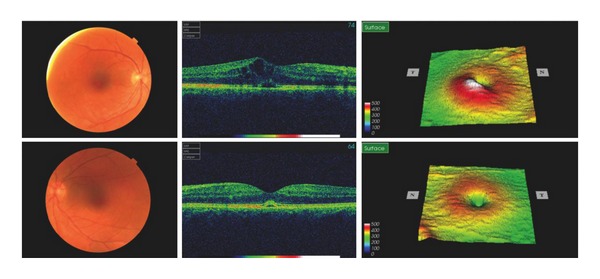
Cystoid macular oedema was resolving after 3 months with minimal subretinal fluid in left eye, although right macular oedema was still significant.

**Figure 3:**
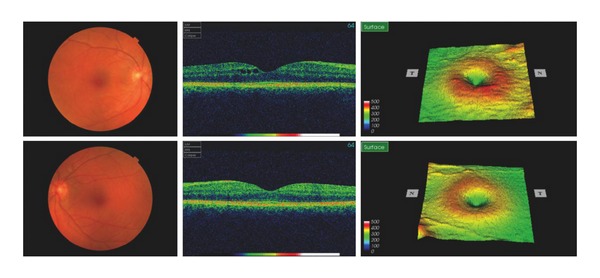
Left macular oedema completely resolved while there was mild residual oedema in right macular after 5 months.

We report a 34-year-old female with a 4-year history of RRMS who developed bilateral cystoid macular oedema after treatment with fingolimod. She had previously two episodes of optic neuritis, which were fully recovered. She otherwise had no other ophthalmic history such as uveitis, diabetic maculopathy, or other retinal disease. Her best corrected vision prior to fingolimod had been 6/7.5 and 6/5. Her MS was previously treated with interferon beta-1b (Betaferon, Schering Health Care) 50 mg subcutaneous injection every other day for two years. As she had 4 relapses between 2010 and 2011 despite interferon beta-1b, oral fingolimod 0.5 mg was started on November 9th, 2011. She developed painless blurred vision 5 days after initiation of fingolimod, which was diagnosed as optic neuritis by her neurologist. Fingolimod was continued for a further 3 weeks. She was reviewed in ophthalmology at that point, when fundoscopy and OCT revealed significant bilateral cystoid macular oedema with subretinal fluid (Figure 1). Her visual acuity then was 6/24 OD and 6/12 OS. Fingolimod was discontinued and she was thereafter followed up in eye clinic monthly. Although the macular oedema and subretinal fluid had improved, they were still present in February 2012 (Figure 2). At this point she started topical 1% prednisolone (Pred Forte) and ketorolac (Acular) three times a day for both eyes. The macular oedema in her left eye was completely resolved by April 2012 with a best-corrected vision of 6/6, whereas there was persistence of minimal cystoid macular oedema in her right eye with a best-corrected vision of 6/12 (Figure 3). 

## 3. Discussion

Fingolimod-associated cystoid macular oedema has been reported in clinical trials looking at fingolimod treatment in both MS and following renal transplantation [4, 5, 7]. A recent case report highlighted development of macular oedema in a 53-year-old MS patient 3 months after initiation of fingolimod treatment [8]. To our knowledge, this is the first report of symptoms of fingolimod-associated macular oedema as early as 5 days after initiation of treatment. This illustrates the importance of early ophthalmology review for patients who experience reduced vision after initiation of fingolimod. Fingolimod-associated macular oedema seems to be dose-dependent with an incidence of approximately 0.5% with 0.5 mg oral dose, which is the currently approved dose [6]. A phase II study reported 4 patients with cystoid macular oedema out of 188 patients on oral does of 1.25 mg or 5 mg fingolimod [9]. In one 24-month clinical trial of fingolimod in MS, 7 out of 429 patients (1.6%) on 1.25 mg fingolimod developed cystoid macular oedema, while none of 425 patients on the lower dose of 0.5 mg fingolimod had this side effect. Five of these 7 cases occurred within 3 months of the start of therapy and 6 cases resolved between 1 and 6 months after discontinuation of treatment [5]. In another large 12-month clinical trial of fingolimod in MS, 4 patients in the 1.25 mg group of 369 patients (1.1%) and 2 patients in the 0.5 mg group of 398 patients (0.5%) were diagnosed with macular oedema. Five of the 6 cases were detected within 4 months and 4 cases resolved within 3 months after discontinuation of fingolimod [4]. It has recently been reported that microcystic macular oedema can be present in MS patients without any prior retinal diseases, which suggests that the breakdown of the blood-retinal barrier and tight junction might also occur in MS [10]. 

The mechanism underlying fingolimod-associated macular oedema is not entirely clear. It has been shown that the S1P receptor not only plays a role in recruiting T lymphocytes, but also in regulating vascular permeability. S1P promotes vascular endothelial cell adhesion to prevent vessel leakage, possibly through S1P and S1P receptor signalling pathways. Fingolimod-phosphate induces degradation of the S1P receptor, which might lead to downregulation of the S1P receptor's role in maintaining vascular endothelial integrity and result in breakdown of the inner blood-retinal barrier [11]. 

Patients on fingolimod should be advised of the possible adverse visual symptoms. It may be helpful to undertake ophthalmologic evaluation prior to treatment and at 3 to 4 months after initiation of the first dose. An Amsler chart can be useful in allowing patients to self-monitor, and urgent ophthalmology review is suggested for patients with visual symptoms especially painless reduced vision. Fundoscopy and OCT should be performed to record progression. Topical steroid and NSAID might be useful to accelerate recovery. Cystoid macular oedema is reversible in most of the cases after early discontinuation of fingolimod. Further studies are required to better determine the incidence and management of fingolimod-associated macular oedema.
